# Ensuring impact of long‐acting HIV therapeutics through multi‐level treatment research: a view from NIH

**DOI:** 10.1002/jia2.26091

**Published:** 2023-07-13

**Authors:** Peter S. Kim, Michael J. Stirratt, Tia M. Morton, Dianne M. Rausch

**Affiliations:** ^1^ Therapeutics Research Program Division of AIDS National Institute of Allergy and Infectious Diseases (NIAID) National Institutes of Health Bethesda Maryland USA; ^2^ Division of AIDS Research National Institute of Mental Health (NIMH) National Institutes of Health Bethesda Maryland USA

1

Long‐acting and extended delivery (LAED) HIV therapeutic regimens represent a revolutionary advance in HIV treatment. However, to fully benefit from this promising advance, LAED regimens must be paired with care delivery innovations that facilitate their access and use. These integrative approaches will require a multi‐disciplinary research agenda that generates additional long‐acting treatment options as well as strategies that support the effective and equitable use of these novel therapeutics.

HIV treatment regimens that require lifetime daily administration present challenges, including treatment fatigue, privacy concerns and suboptimal adherence [1]. LAED regimens can mitigate these challenges for people with HIV (PWH). The first long‐acting injectable (LAI) HIV treatment regimen for maintenance of viral suppression (Cabenuva) entered the US market 2 years ago, and consumer feedback has been overwhelmingly positive [2, 3]. LAI regimens administered on a monthly or bi‐monthly basis may reduce adherence burdens, HIV‐related stigma and privacy concerns that are often associated with daily oral HIV treatment. During a 2022 US Conference on HIV/AIDS session organized by NIAID, NIMH and the Office of HIV/AIDS Network Coordination (HANC), consumer panelists reported that LAI regimens offered them “freedom;” one panelist described LAI treatment as “a dream come true.”

Multiple LAED therapeutics and drug delivery systems are being developed, and some have regulatory approvals for select populations with HIV. The possibilities of LAED regimens can be glimpsed in lenacapavir, a first‐in‐class capsid inhibitor approved for heavily treatment‐experienced patients with multi‐drug resistant HIV‐1 that can be administered subcutaneously every 6 months [4]. Beyond small molecules, broadly neutralizing antibodies (bnAbs) against HIV‐1 are another class of treatments that may offer a LAED treatment option. While still in early development, clinical trials have shown that bnAbs can affect viral suppression with the potential for prolonged treatment durations [5, 6]. Further research is needed to define how to best utilize this new technology for the treatment of PWH, including a better understanding of viral susceptibility and resistance, how to combine bnAbs with anti‐HIV small molecule drugs and/or other bnAbs for best anti‐viral efficacy, and development of assays to predict clinical efficacy among PWH. As these technologies advance, once‐a‐year treatments for HIV may be possible in the future. Continued research to discover and develop promising LAED therapeutic agents will be critical to realizing this aspirational goal.

These new therapeutic options also drive a need for innovations in HIV clinical practice and monitoring. Clinical strategies to enable treatment experienced PWH to benefit from LAEDs, including LAED treatment initiation without prior viral suppression using oral regimens, would have a profound impact on the health outcomes of many PWH, and research towards this end is urgently needed. Research can additionally improve clinical care through the development of tools that facilitate mutual decision‐making between patients and providers on LAED treatment options. Rapid point‐of‐care assays to measure LAED drug levels and HIV viral load would be valuable for monitoring periods of LAED treatment discontinuation or interruption, when drug levels may be sub‐therapeutic and risks for developing HIV drug resistance increase [7]. The pharmacokinetic “long‐tail” that follows the interruption or discontinuation of current LAI regimens will require clinical strategies and oral medication adherence support to prevent the development of drug resistance.

While LAIs open new possibilities for patient care, they have also challenged the operational and workforce models of many HIV clinics. Strategies to overcome these challenges and associated infrastructural hurdles must be developed, tested and applied, including strategies to manage cold‐chain requirements for drug shipping and storage, intensified staffing needs for treatment injection administration and scheduling, and complex billing and reimbursement processes for LAIs [8]. Early experiences with LAI implementation underscore the importance of a multi‐disciplinary, multi‐level research agenda that looks beyond the need for improved therapeutics to focus on supporting people, health systems and communities who need and use these new treatment options.

New LAED therapeutic options should leverage and spur innovations in HIV treatment and service delivery to fully realize their potential impact. Models of healthcare delivery that provide low‐barrier care and integrated services for HIV, chronic conditions and unmet basic needs have improved HIV care engagement and clinical outcomes in populations that experience complex adherence challenges [9, 10]. These person‐centred care models, together with innovations like telehealth that were widely used during the COVID‐19 pandemic, should be leveraged to support equitable access to and delivery of LAED regimens. Demonstration projects in San Francisco and other communities have shown that multi‐disciplinary HIV medical care can be delivered through non‐traditional venues, such as digital tools and mobile medical units [11, 12]. LAED treatment regimens will provide further opportunities for advancing models of community‐based care, including the potential use of pharmacies, mobile vans and home nurse visits to administer LAED regimens to populations with adherence challenges.

Advancing the equitable impact of long‐acting therapeutics will require concurrent consideration of broader social determinants of health, such as intersectional HIV stigma and discrimination, that can impede HIV treatment adherence, retention and viral suppression [13, 14]. Further research is necessary to better understand the mechanisms through which intersectional stigma and other social factors may affect long‐acting antiretroviral therapy access, use and outcomes, which will lead to effective multi‐level interventions to address these issues [15].

LAEDs are a technological advance with the ability to significantly improve patient outcomes for all PWH. However, research is still needed to realize the full potential of this technology. The research needs span the research pipeline from the discovery and development of new therapeutic entities and diagnostic assays to the elucidation of novel clinical strategies that address the many interacting levels of healthcare delivery and patient care. NIAID and NIMH have called for research on these fronts, including innovations in long‐acting regimen development (NOT‐AI‐22‐042), clinical monitoring (PAR‐21‐070), behavioural support interventions (PAR‐23‐060, ‐061 and ‐062) and implementation science (RFA‐AI‐21‐024). Given the need for multi‐sectoral work, academic and commercial partnerships through small business research grants (PA‐22‐176, ‐177, ‐178 and ‐179) could additionally develop and test innovations that may facilitate LAED uptake. Advancing a robust and multi‐level LAED research agenda will better capacitate our ability to overcome inequities in HIV care access and outcomes and bring the HIV pandemic to an end.

## COMPETING INTERESTS

None of the authors has any competing interests.

## AUTHORS’ CONTRIBUTIONS

All authors (PSK, MJS, TMM and DMR) contributed to the conceptualization, writing and editing of this document.

## FUNDING

Nothing to declare.

## DISCLAIMER

The opinions expressed in this viewpoint do not necessarily represent the views of the National Institute of Allergy and Infectious Diseases, the National Institute of Mental Health, the National Institutes of Health, the Department of Health and Human Services or the United States Government.
